# Bridging the Gap: Enhancing Maritime Vessel Cyber Resilience through Security Operation Centers

**DOI:** 10.3390/s24010146

**Published:** 2023-12-27

**Authors:** Allan Nganga, George Nganya, Margareta Lützhöft, Steven Mallam, Joel Scanlan

**Affiliations:** 1 Department of Maritime Studies, Western Norway University of Applied Sciences (HVL), 5525 Haugesund, Norway; 2 School of Education-Sciences, Falmer Campus, University of Brighton, Brighton BN1 9PH, UK; 3 Fisheries and Marine Institute, Memorial University of Newfoundland (MUN), St. John’s, NL A1C 5S7, Canada; 4 Department of Maritime Operations, University of South-Eastern Norway (USN), 3184 Borre, Norway

**Keywords:** maritime security operations center, remote operations center, cyber resilience

## Abstract

Increasingly disruptive cyber-attacks in the maritime domain have led to more efforts being focused on enhancing cyber resilience. From a regulatory perspective, there is a requirement that maritime stakeholders implement measures that would enable the timely detection of cyber events, leading to the adoption of Maritime Security Operation Centers (M-SOCs). At the same time, Remote Operation Centers (ROCs) are also being discussed to enable increased adoption of highly automated and autonomous technologies, which could further impact the attack surface of vessels. The main objective of this research was therefore to better understand both enabling factors and challenges impacting the effectiveness of M-SOC operations. Semi-structured interviews were conducted with nine M-SOC experts. Informed by grounded theory, incident management emerged as the core category. By focusing on the factors that make M-SOC operations a unique undertaking, the main contribution of this study is that it highlights how maritime connectivity challenges and domain knowledge impact the M-SOC incident management process. Additionally, we have related the findings to a future where M-SOC and ROC operations could be converged.

## 1. Introduction

Critical infrastructure sectors have witnessed an increased deployment of Internet of Things (IoT) ecosystems involving sensors and actuators to communicate with physical systems. These IoT ecosystems help optimize and improve real-time operations [1].

The maritime industry, identified as a critical infrastructure sector by the Network and Information Security (NIS-2) directive [2], is a key element in world trade and the global economy, accounting for the carriage of nearly 90% of the world’s goods. Much of this has historically been undertaken on vessels with limited connectivity due to the constraints of operating via satellite networks, whose shortcomings include low bandwidth, high propagation delays, high packet error rates, high initial costs to set up, and finally a high frequency of link breakages [3]. However, the connectivity landscape is quickly changing with the introduction of low-orbit, large-scale communication satellite constellations. The result is rapid digitalization and automation of the maritime domain.

### 1.1. Maritime Digitalization

At the heart of this digitalization wave is the Internet of Things (IoT) which is enabled by a proliferation of sensors resulting in complex digital systems onboard modern vessels and on shoreside port infrastructure. The same digitalization wave is also key to the recent advancements in autonomous vessel development. To ensure their safe operation, autonomous vessels will be ever more reliant on sensor technology for propulsion control, route planning, situational awareness, and collision avoidance [4,5]. The large amount of data being generated by these sensors means that both vessel and port operators have the opportunity to optimize their operations. Some of the innovative applications of maritime big data include remote monitoring of vessels, demand forecasting, predictive maintenance, and digital twin development [6]. A case example is the Port of Hamburg which utilizes data from more than 300 sensors to assist with vessel and port vehicle monitoring. These have been shown to contribute to an overall reduction in idle units which results in lower carbon emissions and increased port operation efficiency [7].

The ever-improving connectivity levels and increasing IoT adoption have led to concerns from both nation-states and infrastructure operators on the need for better cyber resilience of critical infrastructure within the maritime domain, particularly vessels. This is because the ‘air-gapped by default’ status of vessels, necessitated by previously limited bandwidth availability, meant that onboard systems were not designed with cyber security as a consideration. The increased threat-attack surface means that a successful attack could have dire consequences on public safety, economic stability, and environmental concerns [8].

To further reinforce these concerns, in early 2023, the DNV asked 800 maritime professionals what impacts of cyber-attacks they expect to see in the next 1–2 years. A total of 80% of respondents believed that a closure of a major port or waterway, 68% believed a vessel would run aground, 60% believed there would be a collision, and 56% believed there would be a physical injury or loss of life [9,10]. When it comes to autonomous vessels, cyber-attacks have been ranked as one of the top hazards that they face [5].

From a cyber criminal’s perspective, such a threat landscape presents an opportunity to inflict maximum damage with minimal effort. There have been documented cyber-attacks on critical infrastructure in other safety-critical domains, such as the 2010 Stuxnet attack on an Iranian nuclear facility, the 2015 Ukrainian power grid hack, and the recent colonial pipeline ransomware attack in 2021 [11,12]. 

### 1.2. Vessel Connectivity Cyber Risks

**Figure 1:**
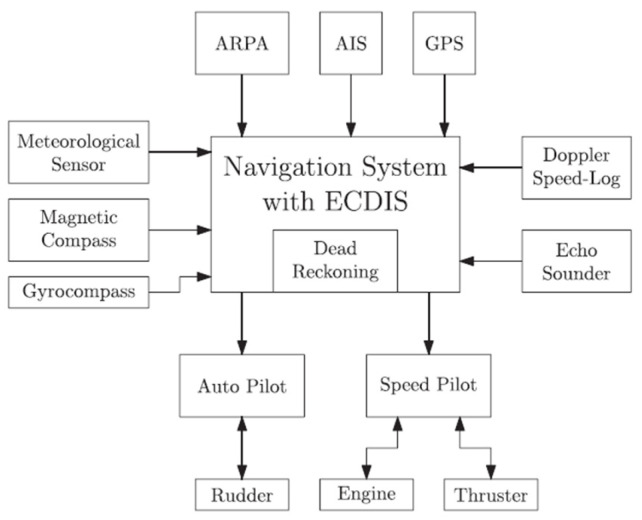
Integrated Navigation System [13].

Improved connectivity has made it a key component of vital vessel functions such as navigation, which has become increasingly computer-aided. Modern vessels are today completely dependent on global navigation satellite systems (GNSS) for correct positioning, navigation, and timing, all complemented by interconnected sensors within what is known as an integrated bridge system. Figure 1, adapted from [13], highlights an overview of a modern-day integrated navigation system (INS).

While the increased adoption of sophisticated communication networks has helped improve navigational situational awareness on the bridge, the downside is that these networks and systems were never designed with security in mind. They have been shown to be increasingly vulnerable to cyber-attacks which undermine the safety of the vessel and its crew [14]. A closer examination both from a research perspective and real-life case examples shows that these concerns are indeed valid.

The study by [15] evaluated the cyber posture of an INS on a roll-on/roll-off ship that was still operational. By using a widely deployed vulnerability scanner, the researchers were able to identify four critical vulnerabilities including an outdated version of the server message block (SMB) service, which was previously exploited by the destructive NotPetya ransomware attack impacting maritime operators such as Maersk [16]. A similar approach of scanning INS components was also utilized by [17]. By scanning the radar systems of two oil tankers, they identified a vulnerable SMB service. Research by [18] evaluated the cyber security risks of key network protocols utilized by maritime vessels. TCP/IPv6, CAN Bus, National Marine Electronics Association (NMEA) 0183, and Automatic Identification System (AIS) were all classified as being at a high risk of Denial of Service (DoS), spoofing, packet sniffing, and relay attacks.

There has been research that has demonstrated how vulnerabilities inherent in the networking infrastructure can be exploited. Research conducted by [19] took advantage of the inbuilt weaknesses in the ASTERIX protocol used for radar data transmission to show how malware can be propagated in the INS. Ref. [20] demonstrated an attack concept exploiting the radar antenna to act as an open door for receiving malicious commands which are then propagated in the INS.

One of the first recorded demonstrations of a Global Positioning System (GPS) spoofing event occurred in 2013 when researchers from the University of Texas caused a yacht to alter its course without the knowledge of the crew onboard [14]. Research by [21] demonstrated a successful case scenario of a GPS jamming attack that led to erroneous AIS position reporting on the radar. The study conducted by [22] successfully highlighted the weaknesses in the internal signaling of the INS by simulating a man-in-the-middle attack that intercepted and manipulated GPS coordinates.

Real-life examples of GNSS spoofing events have also been recorded. In 2017, a ship off the Novorossiysk-Russia shore had its GPS show that it was inland near the Gelendzhik airport. It was later discovered that this problem had affected 20 other vessels in the area, and it was later attributed to a Russian GPS spoofing attack [23]. Around the port of Shanghai in 2018, there were cases of false signals that made it seem that ships were moving in ring patterns. The same phenomenon was also reported at Point Reyes in Northern California, USA where false signals made it seem that ships were moving in ring patterns. In 2019, a UK-flagged oil tanker, Stena Impero, and its crew were seized by the Iranian guard after having drifted into Iranian waters. This was later believed to have been caused by spoofing of the GPS signals. There was also another spoofing event around Elba Island in 2019, where several artificial AIS targets affected the navigation of ships around that area [24].

These examples demonstrate how a seemingly harmless aspect of maritime digitalization, improved connectivity, can result in a significant increase in the threat attack surface and in some cases, lead to crew safety being severely compromised.

This turns the focus to the role of regulation. The maritime domain has been noted to be one heavily dependent on regulation for the improvement of safety [25]. The downside to this approach is a situation where the cyber maturity of the domain lagged due to there being no enforceable regulation until 2017. This left it vulnerable to crippling attacks as was seen in the 2017 NotPetya ransomware attack that significantly impacted the operations of Maersk, a shipping giant [16]. However, the International Maritime Organization (IMO) did pass new cyber requirements that came into force in 2021 [26,27]. The goal of this regulation was to rapidly improve the lagging cybersecurity maturity of the industry. This regulation has been backed by strong industry support from other key maritime stakeholders such as BIMCO [28], DNV [29], Bureau Veritas [30], and the International Association of Classification Societies (IACS) [31,32].

Among other things, the maritime cyber security guidelines passed by the IMO call upon stakeholders to implement measures that would enable the timely detection of cyber threats [26,27]. However, there is a gap in knowledge on the current state of this implementation. This study therefore aims to fill that gap by investigating the industry response to timely cyber threat detection from the perspective of Maritime Security Operation Centers (M-SOCs). Specifically, this study will seek to answer the following research question:

What are the enabling factors and challenges that M-SOCs have faced in their quest to facilitate the timely detection of cyber events?

The findings of the above research question will then be related to a future where autonomous vessel Remote Operation Centers (ROCs) will be more prevalent.

## 2. Background

### Maritime SOC Adoption

Security Operation Centers (SOCs) are an integral part of an organization’s security incident response process, and their effectiveness is dependent upon their visibility into enterprise networks and systems, access to actionable threat intelligence, forensic and analytic capabilities [33].

Their implementation is usually tied to the need to meet business requirements, regulatory requirements, or both. The adoption of M-SOCs by vessel operators has been done to satisfy both business and regulatory requirements. From a business perspective, the increasing incidences of cyber-attacks targeting the maritime domain are a cause for concern. Vessels only make money when they are sailing and so the possibility of a cyber-attack crippling vessel operations would be detrimental to the bottom line. From a regulatory perspective, the IMO cyber security regulation [26,27] calls for the timely detection of cyber anomalies by domain stakeholders. M-SOCs are therefore well-positioned to satisfy both needs, hence their increasing adoption. When it comes to vessel cyber-resilience, M-SOCs aim to monitor both the onboard Information Technology (IT) and Operational Technology (OT) systems in real time for any cyber anomalies.

However, their relatively recent adoption means that the body of research looking into them has only just begun to emerge. Research by [34] proposed four essential blocks for fully capable M-SOCs. The first block defined was the Ship Shore Manager (SSM) whose function was to ensure that all data collected onboard was properly received at the shoreside within the specified bandwidth limits. The Central Processor, which was the second defined block, helped to filter, normalize, and transform all the metadata alerts and logs being received from the ship. The third block, Data Store, was envisioned as a big data engine, while the Bandwidth Manager, the fourth block, was to help monitor the bandwidth configuration for various ships.

With the increased threat attack surface largely acknowledged, one emerging concern is the alarming shortage of qualified cyber security personnel both onboard vessels and shoreside capable of handling not only Information Technology (IT) but also Operational Technology (OT) cyber security [10,35,36]. To compound this problem further, there are currently no formal cyber security training guidelines in the Standards of Training, Certification, and Watchkeeping (STCW) for Seafarers [37].

With the aforementioned cyber skills shortages in the maritime domain, it comes as no surprise that a popular M-SOC research theme has been enhancing M-SOC analysts’ skills, with one study recommending the application of the NIST National Initiative for Cybersecurity Education (NICE) framework [38] to define skills, knowledge, and training objectives of an M-SOC operator [39]. Research by [40] suggested the use of cyber ranges to provide M-SOC analyst training scenarios tailor-made for the maritime domain.

While SOC implementations exist in other critical infrastructure domains, the maritime domain has some unique characteristics that would influence the effective operation of M-SOCs. Poor connectivity at sea meant that for a long time the best defense a vessel had was isolation. This meant that not enough effort was put into designing cybersecure systems. When coupled with the long operational lifespan of vessels, and the cost-conscious nature of the domain [41], we now have a situation where the bulk of the vessels sailing today are heavily dependent on legacy equipment, particularly OT systems. This is a problem for SOCs as a recent SANS report [42] highlighted that the technical integration of legacy systems with modern IT systems was the biggest challenge organizations faced. M-SOCs therefore have to be aware that their visibility into vessel systems and the subsequent effectiveness of their monitoring can be impeded by this technical integration challenge. While it has been noted to be improving [43], offshore connectivity remains a challenge compared to shoreside connectivity. M-SOCs therefore have to prepare for both potential bandwidth limitations and technical integration challenges that may impede their ability to conduct real-time vessel monitoring.

The vessel itself is, at any one time, a fusion of both static and dynamic attributes. The static attributes are most notably represented by the multiple vessel types that exist in operation, each of which is unique in its design [44]. M-SOCs would also have to deal with the fact that vessels are constantly in motion, meaning that contextual information plays a big role both in the type of alerts seen and the volume. The use of contextual information in the incident response process is not limited to M-SOCs but is rather a common routine in SOC operations and has been applied by SOCs in other domains. A 2023 SANS SOC survey [45] highlighted the lack of context as one of the greatest barriers to the full utilization of SOC capabilities. Contextual information in the maritime domain plays a critical role during the incident response process, mainly because the asset being monitored is in constant motion, which exposes it to a greater scope of threats. For example, one of the contextual information categories that could be utilized is the vessel’s geographical location, a dynamic attribute that could be defined as either open ocean, coastal port, harbor, restricted waterways, or inland waterways [46].

Connectivity challenges and dynamic vessel states are some of the key attributes that make M-SOC operations a unique undertaking. Therefore, to fully understand M-SOCs, these maritime-specific attributes cannot be ignored.

#### Maritime Future with Remote Operations Centers (ROCs)

While the adoption of M-SOCs is presently limited to monitoring conventionally manned surface vessels, the future of shipping is progressively moving towards autonomous vessels. Therefore, a discussion on M-SOCs would not be complete without looking to the future where they will be tasked with monitoring autonomous vessels and what the implications of the same would be.

The potential benefits that have been attributed to autonomous vessels include fuel cost savings, crew cost savings, improved crew safety, and a reduction in emissions. The introduction of these autonomous vessels will come with a significant redesign of the fleet operational models, most notably through the introduction of Remote Operation Centers (ROCs) [47]. These ROCs, which have also been referred to as shore control centers [48], would enable greater oversight of vessels and enable shipping companies to have more shore-based control of their assets. Some of the functionalities that have been proposed for ROCs include the monitoring of physical processes onboard the vessel, navigation control, taking over control during emergencies, and implementation of software updates [49]. This presents a context where rich data flows from a vessel are available onshore to operators who may then undertake actions to change the vessel’s state.

The primary concern for humans operating a vessel remotely is their keeping up adequate situational awareness through remote sensing of the state of the vessel [50]. An unmanned ship in a fleet might require support from the operator in the ROC, either due to regulations, mistrust in automation, or automation capabilities. Ensuring connectivity is reliable is pivotal to maintaining adequate situational awareness [48].

More critical challenges the ROC could face concerning maritime security monitoring control systems operation is memory corruption vulnerabilities where the program’s control flow could be hijacked by cyber attackers to execute a random or unreasonable code in the application’s context [51]. The increased data flows arising from the implementation of ROCs expand the threat attack surface which has severe implications for the safe operation of the autonomous vessel. While not comprehensive, such scenarios highlight that there is a natural overlap for the M-SOC when it comes to defending both the vessels and the ROC from cyber-attacks. While currently limited, a body of research has emerged looking into how cyber security can be better managed in an ROC. For example, recent research investigated how cyber alerts can be effectively presented to ROC operators in a way that does not negatively impact their overall situational awareness, yet still elicits a sufficient response [52]. Potential cyber-related risks in the ROC have also been identified including confidentiality breaches, data tampering, and the loss of connection availability [5].

For autonomous vessels more than conventionally manned ships, connectivity, and efficient ship-to-ship and ship-to-shore communication are key components for their safe operation [53]. Factors to consider include the speed and reliability with which the data generated from sensors and cameras can be transferred between the vessel and the ROC to enable real-time situational awareness and handling for operators [54]. This is an area that has increasingly received research attention. Research by [55] conducted a risk assessment of hazards that would impact the safe operation of autonomous vessels. Some of the hazards that featured in their final ranking included system failure due to a breakdown of the communication link and a system failure due to the jamming or spoofing of AIS and GPS signals.

When it comes to managing the efficient flow of data in the communication channels, Ref. [56] for example looked into spectrum requirements for the control and communication of autonomous vessels. Another study by [57] sought to mitigate time delays in ship-to-shore communication by applying an augmented state cubature Kalman filter (AS-CKF).

Concerns about insufficient link capacity were also raised by [58]. Their study began by highlighting weaknesses in the vertical handover mechanism, which is a common approach that has been suggested as a way to mitigate the link capacity problem. This led them to propose a ship-to-shore communication framework (SSCF) whose characteristics included the ability to be self-adaptive, source-agnostic, lossless, and secure. They then built a network optimizer that met the SCCF framework principles. Their evaluation to investigate whether it would satisfy both ROC and M-SOC data requirements turned out successful, even though it will need future fine-tuning to remove excess noise in the data.

When it comes to the actual operation of an autonomous vessel, research by [49] developed an architecture that is well representative of how these vessels will be managed. Their architecture had a strong focus on the vital role played by connectivity as an enabler for autonomous vessel operation. One standout feature in their architecture was the incorporation of a connectivity manager as one of its core components. Its function was to help control the information flow between the vessel and the ROC [59]. Research by [60] sought to investigate how both satellite and terrestrial connectivity can be integrated to facilitate autonomous vessel operation. Their architecture similarly utilized a connectivity manager whose functions included prioritizing and allocating data to the available communication channels, ensuring there is enough capacity for the data to be transmitted, and that the data reaches its destination within the pre-defined latency requirements.

This functionality is very similar to the Ship Shore Manager (SSM) defined by [34] whose function was to ensure that all data collected onboard was properly received at the shoreside within the specified bandwidth limits. Here, we can see that there is an overlap of components that provide similar functionality for both M-SOCs and ROCs. Thus, the development of ROCs could be informed by the progress that has been made within M-SOCs just as it can be informed by existing remote operation centers used within other critical infrastructure sectors. This should be done while considering the specificity of the challenges faced by the maritime domain.

Contextual information is also relevant when it comes to the safe operation of autonomous vessels in the ROC. One study by [61] introduced the concept of the operational envelope, which defines conditions and scenarios under which an autonomous vessel is meant to optimally function, ship system context, voyage and operational phases, and the division of responsibility between the operator and vessel. It also includes what they defined as a state space, which includes the environment (traffic density, wind, temperature) and the system (sensors, engine state, ship stability). Additional research that has utilized the concept of the operational envelope includes [62,63].

## 3. Method

To answer the research question, we applied criterion sampling [64] in the selection of our participants who had to be working in either an in-house M-SOC or managed security service providers (MSSPs). Five M-SOCs agreed to have their analysts take part in our interviews. These M-SOCs had all been in operation for less than five years. Their organizational profile is highlighted in Table 1.

The study obtained ethical approval from the Norwegian Center for Research Data with the assigned project number 241816.

With the help of an interview guide, the nine participants were individually interviewed between October 2022 and February 2023. Each interview was conducted in English and lasted 1 h on average. Seven interviews were conducted and recorded on Teams [65] and Zoom [66] platforms, while the remaining two took place physically on the premises of the respondents. In this case, an audio recorder captured the interview. Microsoft 365 [67] was used to transcribe the interviews. The coding process was done using Microsoft Excel [68], which also helped track emerging themes.

The analysis of the interview data was informed by grounded theory, which has three phases: open, axial, and selective coding [69]. The interview data first goes through open coding, where responses are grouped according to similar words, phrases, or concept indicators. The next phase, which is axial coding, involves establishing relationships between the open codes previously developed. The output of this phase is the development of sub-categories. Finally, selective coding involves developing a core category that best links all the sub-categories developed in the axial coding phase [69]. Saturation was determined through a code frequency count, which involved establishing the point at which new codes stopped emerging from successive transcripts. Due to the homogeneity of the interview participants, we achieved saturation upon the completion of seven interviews [70]. All analysis and recording of memos were done by the first author.

## 4. Results

**Figure 2:**
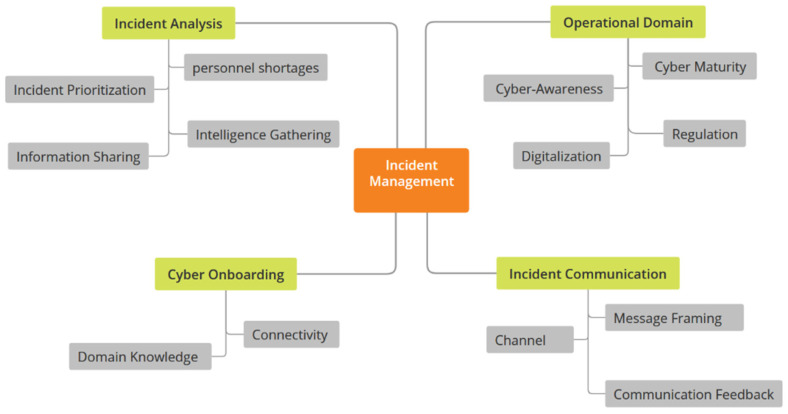
Thematic Map.

Following the conclusion of the selective coding phase of data analysis, incident management emerged as the core category. This means that the discussions with the M-SOC analysts all pointed toward how they conduct incident management and the factors that influence it. This core category was supported by four other categories, namely incident analysis, cyber onboarding, the operational domain, and incident communication. Table 2, Table 3, Table 4 and Table 5 provide a summary description of each category and the respective sub-categories. Additionally, Figure 2 visualizes the categories and sub-categories.

Discussion of the findings will center on the factors that make M-SOC monitoring different from what is experienced in other critical infrastructure sectors. As highlighted in the literature review, connectivity, and varying vessel states are the standout differences that make monitoring in the maritime domain vastly different from other domains. The cyber-onboarding category, constituting connectivity and domain knowledge, aptly captures the concerns around these differences.

### Cyber Onboarding

The onboarding process is a critical step for any SOC. Moreso for an M-SOC because they are at any one time having multiple vessel types being monitored. An understanding that these are bound to have differences in their monitoring requirements is an important first step.

“Yes, there are different systems that you can find on board super yachts that you will not find on commercial ships and vice versa. But that is true for any type of vessel. Another thing is that in many cases they are not unified. So, company X could have 20 vessels, but each one could be completely different from the other. So not always there you will see a unified infrastructure or anything like that.”

(P4)

However, while these differences make monitoring more challenging, it was also highlighted that it could benefit the domain by making propagating vessel cyber-attacks more difficult.

“We believe that an attack will first go to other sectors than the maritime sector. This is because, in the maritime sector, every vessel is unique, so it’s more difficult to attack. An attacker would have to tailor-make everything, so I think that is part of the protection as well. It does not mean that we can continue that down that path. But currently, I think the maritime sector is more protected than a lot of other kinds of sectors.”

(P8)

Connectivity challenges have for a long time been known to plague the maritime domain, particularly in the deep sea. This will be an even more pressing concern when a future with autonomous vessels is considered. It was therefore not a surprise when connectivity emerged as one of the key considerations during the cyber onboarding process. Additionally, not all respondents had managed to set up real-time monitoring due to connectivity challenges:

“The connectivity depends on how far they operate from a coast. Offshore supply can be either way. They can either have 4G or even be connected to a platform that has a wired connection, but if they’re crossing an ocean, they do not have connectivity.”

(P7)

“Because of the current connectivity state of the vessels, we do not have the luxury to monitor our vessels 24/7. However, because this will be the case in the near future, we have started considering how to integrate some cybersecurity solutions with the SOC service to generate reports that will give us a picture of what is happening in our fleet.”

(P9)

For the M-SOC that had managed to set up real-time monitoring, there were still concerns about how much the network could sustain and if they had to prioritize only critical systems:

“I think for us we are very concerned on how much connectivity there is when we are setting up. For example, if they say they only have 256 KB bandwidth, then we have to prioritize which systems are more important. The servers might be more important than the client systems. So, we monitor those areas that we make a prioritization on.”

(P8)

It was, however, highlighted that the implications of a stable connection would be an increased throughput which can be easily managed by the M-SOC if their triage process has been automated.

“It does not affect the work we do. It does affect the output because more connectivity means more data going through the system, more threats that could be detected, and more false positives that need to be detected and ignored. The main change is the rate of data.”

(P4)

Additionally, our respondents highlighted that their monitoring approach presently involves focusing on systems that are already internet-facing. The rationale behind this is to avoid increasing the vessel threat attack surface:

“What we see is that we do not want to monitor things that are not connected to the Internet. So, if something is offline, it remains offline. We do not want to monitor it, so instead we just prioritize what you are going to connect to the rest of the world.”

(P7)

Crucially for operational technology (OT) systems onboard the vessel, which are increasingly coming online, a stable connection is always preferred for efficient monitoring:

“So, for IT, I do not think it’s that much of a problem because if the vessel is offline, you cannot hack it. The problem comes with OT because you want to have constant monitoring and when you lose the connection, you do not know what’s going on. You want to have equipment on board the vessel that can monitor it still and then pass the alert when the link is up again.”

(P8)

Vessel differences from a connectivity perspective were also apparent in the way they were set up to maximize bandwidth usage. Superyachts were mentioned frequently as one of the vessel types that have significantly higher connectedness compared to most other vessel types:

“The loads can be different because usually, super yachts are more connected like bandwidth is used much higher rates over there.”

(P4)

“Well, from our point of view, it’s very different because super yachts have a lot of gadgets and new technologies like IPTVs, so they have great connectivity. They have huge bandwidth of vsat and whatever they need.”

(P6)

However, what has also been seen is that the setup of the IT systems is usually largely similar even across different vessel types. This could probably point to the OT setup being the differentiating factor.

“But right now, we are monitoring a few passenger vessels: medium and long-range ferries, and a lot of offshore support vessels. Their IT onboard is often built up very similarly such as e-mail, end-users, etc.”

(P7)

When it comes to the implications of having a more connected domain, there was a consensus that it will certainly result in more attacks but there was also confidence in the ability to handle them:

“So basically, yes as regards the connectivity enhancements in the near future, there will be of course more cyber-attacks occurring in vessel digital infrastructure, but I believe there are solutions that can support the cyber security part of the operation of the vessels.”

(P3)

Another perspective that emerged from the discussions was how crucial context plays a role during their monitoring activities and in understanding the alerts that they were seeing on the dashboard:

“So, the system is context aware. We understood early on that the difference between a ship and an office, shockingly enough, is that it’s moving, but not only that but it has several operations states. So, for example, when the ship is in the port, usually the activity is very high because that is where most work is being done. And when the vessel is sailing the activity is a lot less.”

(P4)

“In our system specifically, have several anomaly detection mechanisms on several level layers and context is part of the information fed. So, for example, where is the ship at and other contextual information depending on how we connect.”

(P4)

“In many aspects, you need to know what’s going on in the vessel itself. Again, another example is if the ship is in the port, you could see an indication of unwanted activity. But that could be just the consequences of the maintenance because some guy is connecting something.”

(P8)

“The context is key to rapidly understand if we have a false positive or a true positive.”

(P5)

Multiple crucial perspectives emerged during the discussion. With regard to connectivity, low bandwidth concerns meant that prioritizing critical systems was something that warranted further consideration by the M-SOCs. Additionally, it was highlighted by one respondent that they only monitored systems that were already internet-facing to keep the threat attack surface to a minimum. Finally, context awareness was critical during the monitoring process, with geographical location emerging as one of the most utilized sources of contextual data.

## 5. Discussion

This study sought to understand the enabling factors and challenges faced by M-SOCs in their quest to facilitate the timely detection of cyber events. The analysis of the results focused on the factors that make M-SOC operations different from traditional SOCs in other critical infrastructure sectors. These factors are the level of connectivity and the dynamic vessel states that influence contextual information. The subsequent discussion will therefore be guided by these factors. Additionally, the relevance and transferability of these findings for future ROCs will also be explored.

### Connectivity and Domain Knowledge

Effective M-SOC operations in any critical infrastructure domain are highly dependent on having good visibility into all the assets. A 2022 SOC performance report highlighted a lack of visibility as the biggest factor contributing to the ineffectiveness of SOCs [71]. While connectivity is a major contributor to having good visibility of all the assets that are planned for monitoring, its criticality is magnified when monitoring offshore assets as compared to onshore assets. High bandwidth connectivity is almost always a guarantee for most onshore locations. The same, however, cannot be said for offshore locations. For a long time, offshore locations, particularly in the deep sea, have been characterized by high initial set-up costs, low bandwidth even for the paid-for service, high propagation delays, and often unsteady connection with frequent link breakages. This is the environment that M-SOCs have had to deal with, particularly when vessel monitoring is considered.

These concerns were not lost on our respondents, some of whom highlighted that they were yet to operationalize real-time monitoring due to bandwidth limitations. The degree of vessel connectivity was also one of the concerns that they had to factor in during the onboarding process so that they could establish the service offering they could provide to their customers. Closely related to this discussion was the need to prioritize critical systems on board if the bandwidth could not support monitoring all systems.

Incident prioritization as a critical SOC process is not a new concept. Due to the high volume of alerts often seen in the SOC, prioritization has been suggested as one of the ways that can help analysts effectively handle this sea of data. For example, a study by the Naval Research Laboratory defined some prioritization parameters which included priority queuing, the host importance, incident criticality, asset criticality, and connectivity between hosts [72]. Strategy 5 in MITRE’s 11 strategies of a world-class SOC report talks about the need to prioritize the incident response process [73].

While M-SOC analysts would stand to benefit from prioritizing alerts due to the potentially large volume of incoming data, the type of prioritization strategy of most concern given the current bandwidth limitations would be determining the critical systems on the vessel and focusing monitoring on those. This is, however, not a simple task as all systems could potentially be considered critical for the safe operation of the vessel. One of the maritime cyber security guidelines that M-SOCs can use as a guide in determining critical systems onboard the vessel is the Bureau Veritas rules on cyber security for the classification of marine units (NR 659) [30]. These guidelines have established a criticality assessment whose function is to group IT and OT equipment into three levels of criticality, namely harmless, significant, and critical. The criticality assessment has multiple inputs, among them being the system connectivity, which refers to its interconnectedness to other systems onboard the vessel. These guidelines are an example of maritime-specific attempts at classifying the critical systems onboard the vessel.

Once connectivity has been established and monitoring has begun, there emerges another challenge of determining which of the alerts are true positives and those that are false positives. The use of contextual information has been proposed as one of the efficient ways of dealing with this challenge [74] with the same also being highlighted by our respondents. Within the M-SOC context, geographical information emerged as the most prevalent type of contextual information used by analysts to help determine whether an alert was a true positive or a false positive. As was highlighted in the literature review, there have been previous incidences of vessels experiencing GPS and AIS spoofing attacks by being near geopolitically sensitive navigational areas [23].

However, geographical information is not the only type of contextual information that M-SOCs can utilize. Research by [75,76] developed a vessel surveillance anomaly detection framework. In it, they defined the two main categories of anomalies, namely dynamic kinematic and dynamic non-kinematic anomalies. Dynamic kinematic anomalies captured were the vessel course, speed, maneuver, reporting, and location. Dynamic non-kinematic anomalies included the ports of call (departure and arrival), cargo list, ship signature, crew list, and passengers. While not comprehensive, these are additional data points that M-SOCs can consider using to help refine their incoming alerts.

#### Relevance for Future ROC

Having up-to-date and actionable knowledge of the environment that is to be monitored is crucial for the successful operation of an ROC, just as it is for M-SOCs. Achieving this is dependent on having an effective communication network. The level of communication required to support autonomous ships would need to be bidirectional, scalable, secure, and supported by multiple systems to ensure there is redundancy and minimal risk. The connectivity solution has to guarantee sufficient communication link capacity for sensor monitoring and remote control [60]. A review of the literature highlights more effort being dedicated to resolving autonomous vessel connectivity challenges. In the literature review, we highlighted research looking into spectrum requirements and mitigation of communication delays [56,57]. Ref. [58] built a network optimizer based on their ship-to-shore communication framework (SSCF), which was a proof of concept on how a future ROC and SOC would be able to optimize their data flows for efficient operation. The concept of a connectivity manager to help manage data flows was also a standout feature in the literature on how to improve connectivity for autonomous vessel operation [59,60]. We are therefore of the opinion that much of the research on how to optimize connectivity for autonomous vessel operation is applicable and can be carried over to M-SOC operation.

When it comes to the use of contextual information, we saw that geographical information is the most commonly utilized for M-SOCs. Research utilizing the concept of operational envelopes, which aims to enhance situational awareness for ROC operators, has similarly used geographical information as part of the contextual data [61,62,63]. It has, however, been enriched with both environmental (traffic density, wind, temperature) and system data (sensors, engine state, ship stability). There are clear overlaps in the type of contextual data utilized by both ROCs and M-SOCs further reinforcing how both entities can closely collaborate in the efficient execution of their operations.

## 6. Methodological Discussion and Limitations

Most of the SOC studies that we reviewed have used respondents from diverse domains [77,78,79,80]. While this can help provide a broad perspective of the challenges SOCs are facing, their results cannot be said to be representative of any specific domain. By only focusing on M-SOC respondents, we can claim that our findings are representative of the maritime domain.

This study also had to contend with the small sample size for several reasons. The first is that M-SOC adoption has been a recent undertaking. As such, there was a very limited number of either in-house M-SOCs or MSSPs we could contact. Additionally, the sensitive nature of the work that these entities are tasked with performing meant that some of the M-SOCs contacted were uncomfortable participating in the study. This further reduced the pool of potential respondents. Similar SOC studies in other domains have had to contend with participant pool challenges but have managed to obtain meaningful results despite the small sample sizes [78,80,81]. However, by utilizing semi-structured interviews, we were able to obtain sufficient depth in the responses.

Constant comparison guided the data collection and coding process, which helped minimize the impact of bias. To ensure quality, as viewed from the interpretive lens, was maintained, the coding process was documented by the primary author through memos. These were reviewed by the co-authors to ascertain the representativeness and confirmability of the findings. Visualizing the output of the coding process through Table 2, Table 3, Table 4 and Table 5 and Figure 2 helped ensure the dependability of the study. The findings on connectivity and contextual information, as presented, are specific to the maritime domain.

## 7. Conclusions

Increasingly disruptive cyber-attacks in the maritime domain have led to more efforts being focused on enhancing cyber resilience. With the interest in vessels leveraging more automation and the likely adoption of ROCs, the impact of cyber-attacks could grow in the coming years. In response to a regulatory need for timely detection of cyber events, vessel operators have increasingly adopted M-SOCs. Their relative newness led us to develop this study, which sought to better understand both enabling factors and challenges that impact the effectiveness of their operations. Interview data was collected from M-SOC analysts. Informed by grounded theory, we analyzed the interview data of nine M-SOC analysts, which led to the emergence of incident management as the core category. Subsequent discussion of the results focused on the challenges that make M-SOC operations unique from those of other critical infrastructure domains.

From a connectivity perspective, we saw that bandwidth concerns meant that M-SOCs had to consider prioritizing onboard systems that they felt were critical. This differs from the prioritization seen in other SOCs, which is almost always due to an overload of data. However, significant connectivity improvements will shift prioritization needs towards dealing with alert overload. Dynamic vessel states also meant that geographical positioning was the most utilized contextual information when it came to filtering out false positives from true positives. The findings are also very relatable to a future with ROCs, which we saw face the same challenges of connectivity and utilization of contextual information for situational awareness.

This research has been able to show how connectivity and contextual information, as applied in the maritime domain, make M-SOC operations different from other SOCs. We have also been able to show how these relate to ROCs and the synergies that exist between the two entities. In a future where both M-SOC and ROC operations could be converged, the authors recommend that future studies build on these findings.

## Figures and Tables

**Table 1:** M-SOC Organizational Profile.

Organization	Operating Model	Staff Count	Fleet Size	Fleet Type	Interview Participants
M-SOC 1	MSSP	10	500	Tankers, Floating Production Storage and Offloading (FPSO)	P1, P2, P3
M-SOC 2	MSSP	15	200	Superyachts, cruise ships, container vessels	P4
M-SOC 3	MSSP	10	300	Superyachts, passenger, and container vessels	P5, P6
M-SOC 4	MSSP	9	150	Container and passenger vessels	P7, P8
M-SOC 5	In-house	4	60	Tankers	P9

**Table 2:** Incident Analysis Category and Sub-Categories.

Category	Description
Incident Analysis	Process of investigating the root cause of the alert.
***Subcategory***	***Description***
Personnel Shortages	Relates to the impact of M-SOC staff shortages on the incident analysis process.
Incident Prioritization	How M-SOC analysts rank the criticality of incoming alerts.
Intelligence Gathering	The process of obtaining additional information related to the alert that would assist with its analysis.
Information Sharing	The ease with which cyber threat information is disseminated within the maritime domain which would assist in timely incident analysis.

**Table 3:** Operational Domain Category and Sub-Categories.

Category	Description
Operational Domain	The industry within which the M-SOC operates, maritime in our case.
***Subcategory***	***Description***
Cyber Maturity	Maritime stakeholders’ investment in cyber security preparedness, recovery, and business continuity
Cyber Awareness	Cyber awareness levels of crew onboard the vessel and shoreside personnel.
Regulation	Existing maritime cybersecurity regulation.
Digitalization	The level of technological adoption in the maritime domain.

**Table 4:** Cyber Onboarding Category and Sub-Categories.

Category	Description
Cyber Onboarding	Ensures client monitoring requirements are fully captured and incorporated into the M-SOC monitoring platform.
***Subcategory***	***Description***
Connectivity	The degree to which the vessel has sufficient internet access and bandwidth to facilitate real-time monitoring.
Domain Knowledge	Contextual knowledge regarding vessel operations such as key onboard systems and sailing routes.

**Table 5:** Incident Communication Category and Sub-Categories.

Category	Description
Incident Communication	The procedures involved in alerting the crew onboard the vessel on the cyber threat alert.
***Subcategory***	***Description***
Message Framing	How the threat information is communicated to elicit a response from the recipients (crew).
Channel	The medium used by the M-SOC to convey the cyber threat alert to the crew onboard the vessel.
Communication Feedback	The learning phase for the M-SOC where the crew and M-SOC work on improving the incident communication process.

## Data Availability

The data presented in this study is available on request from the corresponding author. The data is not publicly available due to privacy and ethical reasons.
